# A new functional Hip Scoring System compatible with Asian Lifestyle

**DOI:** 10.12669/pjms.38.3.5758

**Published:** 2022

**Authors:** Javeria Junaid, Anisuddin Bhatti

**Affiliations:** 1 Dr. Javeria Junaid, MBBS. Trainee Medical Officer, Department of Orthopaedic & Spine Surgery, Dr. Ziauddin University Hospital, Clifton. Karachi, Pakistan; 2 Dr. Anisuddin Bhatti, FCPS. Consultant Orthopaedic & Paediatric Orthopaedic Surgeon, Department of Orthopaedic & Spine Surgery, Dr. Ziauddin University Hospital, Clifton. Karachi, Pakistan

 The goals of treatment in children with hip problems are to create normal anatomy of the proximal femur and acetabulum and maintain that anatomy to allow normal development of hip function. With experience and greater understanding of the outcomes, surgical recommendations frequently change. These surgeries are complicated, and the outcome has a direct correlation with experience.^1^ Therefore, value of being able to objectively evaluate a patient’s functionality postoperatively is unmeasurable. Several universally accepted popular evaluation criteria for pre- & post-surgical hip evaluation includes Merle, D. Aubigne R (1949), Larson’s Iowa hip Score (1963), Harris Hip Score (1969), and D’Arcy, J (1979) systems cited by Brand RA^2^ are meant for pre & post-surgical hip evaluations. They serve as the basis for objective analysis of postoperative hip functionality, endurance, walking ability, siting on chair seat, and driving, along with Range of Motion (ROM) measurements. All these methods are meant for adult hip and are of insignificant value for use in Paediatric hip evaluation.

 In Paediatric pathologies such as, Developmental Dysplastic Hips (DDH), Perthes’, Slipped Capital Femoral Epiphysis, Femoral neck fractures and post inflammatory states, the pre- and post-surgical comprehensive evaluation plays a significant role in progressive development of hip. The existing Paediatric hip clinical evaluation systems includes Ferguson (1931) Severin (1950), Chuinard (1963) McKay (1974), Trevor (1975) cited by Bhatti A et al.^3^ and Modified MacKay criteria and modified Harris hip score (2012) cited by Abdullah EAH et al.^4^ McKay, Trevor, and Modified MacKay criteria and modified Harris hip score are the most frequently used methods. The parameter used by these methods include, presence of pain, limp, stability in the supine position, Trendelenburg sign, deformity, ROM, functional limitations, and endurance. The functional limitation evaluated includes “as described by the patient” that “partially limited or severely limited”.^5^ These functional limitations or types of functions have never been categorized or explained before.

 All these systems have been designed with needs of western lifestyle in mind. Whereas the eastern (Asian) population have some unique sitting habits to sit on floor with positions of Squat (Crouch Sitting), Palthi (Cross leg sitting) and Tashahhud (Kneeling) as shown in Fig. A-C, that has never been addressed in these methods of evaluation. These sitting postures in Asian lifestyle are accustomed and used every day for eating, prayers, work on floor and maintain bowel habits. To adopt these postures, place a huge exertional demand on postoperative hip is required, along with a good stability, balanced muscle power, supple pain free hip, knee, and trunk. The currently available scoring systems reveal significant limitations to evaluate patients’ endurance and ability to attain these exertional activities, commonly practiced every day in eastern lifestyle and need of the day for children for assimilation into society and not feeling like an outlier. Failure to achieve those postures produce a significant emotional and psycho-social burden to the parents, patient, and community.^3^ Henceforth, it is thus a need of time to do develop a method, that better suits to evaluate patient’s limitation to achieve postures of Squat, Palthi and Tashahhud. Bhatti A et al introduced a new system that evaluate post-surgical reduction of DDH while patients perform their accustomed sitting habit, as the Bhatti’s Functional Scoring System (BFSS).^3^,^6^,^7^ These sitting habits are further categorized in three types as per limitations exhibited and thereafter scored as Excellent, Good, Fair & Poor with combinations of types of siting habit. The author / designer has further renamed BFSS as “Bhatti’s Functional Hip Score” (BFHS) and included types of sitting habits and all other parameters of limp, discomfort, endurance, and Range of Motion in the modified table as in table1 and explained in Fig.A-C.

**Table-I:** Bhatti’s functional hip score (Modified 2021).^3^,^6^,^7^

Sitting Habit	Type-I	Type-II		Type-III
Squat (S)	Able to squat comfortably.	Able to squat with heel raised. Take support by hands on floor.		Difficult to sit in Squat without support, hips raised from floor. Difficult to maintain. Easy to sit in chair but difficult to prop-up.
Palthi (P)	Able to sit in Palthi comfortably.	Able to sit in Palthi with knee raised from floor for <45°. or take support by hands on floor or hold the knees.		Difficult to sit in Palthi without support, hips raised from floor. Difficult to maintain. Keep knee raised from floor over 45°
Tashahhud (T)	Able to sit in tashahhud comfortably.	Able to sit in Tashahhud. Take support by hands on floor or adopt W-sitting.		Difficult to sit in Tashahhud without support, hips raised from floor. Difficult to maintain. Easy to sit in chair but difficult to prop-up.
Endurance	Good	Fair		Poor
Discomfort, & limp	None	Mild		Significant
Range Of Motion	Full ROM	Partially limited		Severely Limited
BFHS Score	Excellent	Good	Fair	Poor
	SI+PI+TI	SI+PII+TI, SII+PI+TI	SII+PII+TI, SII+PII+TII.	SIII+PIII+TI.orTII. SIII+PIII+TIII.

**Fig. A-E:**
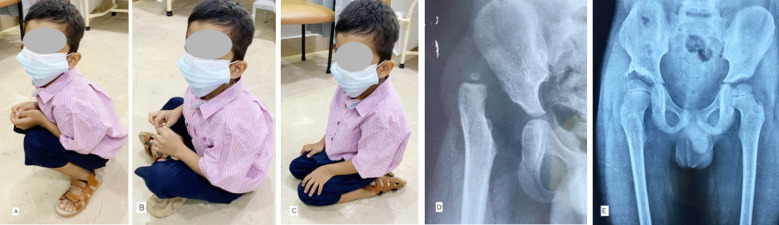
A 30-month-old boy, 30 months after open reduction and Pemberton’s Pelvic osteotomy of right side DDH. Siting at ease in position of Squat Type-I (A), Palthi Type-I (B) & Tashahhud Type-I (C). BFHS Score Excellent. Pre-operative Xray show Tonnis IV dislocation (D), Post-operative Xray show Severin class1a (E). Clinico-radiological score Excellent.

 The limitations exhibited by patients while adopting these postures are documented, recorded in video, and photographed. The video recording and photography without physical contact of the evaluator, significantly improve compliance of the patient, save time of concerned surgeon. The video can also be recorded easily at home by the parents, that provide a bias free, remote evaluation for a long-term follow-up and research.

 The BFHS is recommended to be used after a minimum one-year post-operative duration. The duration that is mandatorily required to get back pain free, supple mobile hip joint with remodeling of articular surfaces, and relief of stiffness. By this time patient regain muscle strength, stability, and balance to have an endurance to perform these exertional activities. The effective use of BFHS after a year and quarterly thereafter helps in confidence build-up amongst parents and surgeon, with a real time demonstration of progressive development of hip/hips.

 A pilot study of comparison between “outcome of Bhatti’s vs Mackay & Ferguson scoring system” in post-surgical reduction of DDH^3^ revealed comparatively similar results in terms of presence of pain, limp, Trendelenburg sign and endurance. Whereas BFSS was found superior scoring system to evaluate functional limitations of Asian lifestyle, having better compliance, less time consuming, reliability and accuracy better than MacKay & Ferguson with kapa value 100.0.

 Moreover, the combination of BFHS with Severin’s radiological classification^8^ provides a comprehensive progressive clinico-radiological evaluation of the paediatric post-surgical hip/hips (Fig.A-E). That is significantly compatible with Eastern (Asian) lifestyle as well.

## Authors’ Contribution:

**JJ:** Literature search, manuscript writing.

**ANB:** Concept, design, research, innovation of BFHS, manuscript review & permission to publish.
